# Challenges and Opportunities for Clinician Implicit Bias Training: Insights from Perinatal Care Stakeholders

**DOI:** 10.1089/heq.2023.0126

**Published:** 2023-09-13

**Authors:** Sarah B. Garrett, Linda Jones, Alexandra Montague, Haleemat Fa-Yusuf, Julie Harris-Taylor, Breezy Powell, Erica Chan, Stephen Zamarripa, Sarah Hooper, Brittany D. Chambers Butcher

**Affiliations:** ^1^ Philip R. Lee Institute for Health Policy Studies, University of California, San Francisco, California, USA.; ^2^ California Preterm Birth Initiative, University of California, San Francisco, California, USA.; ^3^ UCSF-UC Law Consortium on Law, Science & Health Policy, University of California College of the Law, San Francisco, California, USA.; ^4^ Independent Researcher and Community Advisor, San Francisco, California, USA.; ^5^ University of Colorado Anschutz Medical Campus, Aurora, Colorado, USA.; ^6^ Department of Human Ecology, University of California, Davis, California, USA.

**Keywords:** stakeholder engagement, implicit bias training, maternal health, health equity intervention, community-based participatory research, qualitative research

## Abstract

**Introduction:**

In an attempt to address health inequities, many U.S. states have considered or enacted legislation requiring antibias or implicit bias training (IBT) for health care providers. California's “Dignity in Pregnancy and Childbirth Act” requires that hospitals and alternative birthing centers provide IBT to perinatal clinicians with the goal of improving clinical outcomes for Black women and birthing people. However, there is as yet insufficient evidence to identify what IBT approaches, if any, achieve this goal. Engaging the experiences and insights of IBT stakeholders is a foundational step in informing nascent IBT policy, curricula, and implementation.

**Methods:**

We conducted a multimethod community-based participatory research study with key stakeholders of California's IBT policy to identify key challenges and recommendations for effective clinician IBT. We used focus groups, in-depth interviews, combined inductive/deductive thematic analysis, and multiple techniques to promote rigor and validity. Participants were San Francisco Bay Area-based individuals who identified as Black or African American women with a recent hospital birth (*n*=20), and hospital-based perinatal clinicians (*n*=20).

**Results:**

We identified numerous actionable challenges and recommendations regarding aspects of (1) state law; (2) IBT content and format; (3) health care facility IBT implementation; (4) health care facility environment; and (5) provider commitment and behaviors. Patient and clinician insights overlapped substantially. Many respondents felt IBT would improve outcomes only in combination with other antiracism interventions.

**Health Equity Implications:**

These stakeholder insights offer policy-makers, health system leaders, and curriculum developers crucial guidance for the future development and implementation of clinician antibias interventions.

## Introduction

The United States has poorer maternal health outcomes than peer countries and substantial racial and ethnic inequities among them.^1–7^ Black women and birthing people in the United States, for example, are approximately three times more likely to die from a pregnancy-related cause than white women and birthing people, and experience significantly higher rates of severe maternal morbidity (e.g., preeclampsia, preterm birth) and neonatal mortality.^7–10^ This elevated risk, deriving from historical and contemporary manifestations of structural and interpersonal racism,^11–13^ exists across the socioeconomic spectrum.^14–16^ Women and birthing people from historically oppressed populations in the United States report higher rates of mistreatment during pregnancy and birth than white individuals, and report disrespectful, disempowering, and coercive interactions with perinatal care providers.^17–22^ These unjust and preventable^23^ inequities persist even in states where aggregate outcomes have improved.^21,24–27^

Recognizing the threat of implicit bias to patient care and outcomes,^28–33^ 25 states and Washington DC have since 2019 introduced, and 6 have passed, legislation requiring implicit bias training (IBT) for health care workers.^34^ The California legislature enacted the “California Dignity in Pregnancy and Childbirth Act,” effective January 2020 (“The Act”).^35^ Among its core components is the requirement that all clinicians providing perinatal care at hospitals, licensed alternative birth centers (ABC), or primary care clinics providing ABC services undergo evidence-based IBT every 2 years, addressing 10 specific foci (Supplementary SA).^35^ Many California facilities have directed providers to new or existing self-administered online trainings to fulfill these requirements.^36^ The Act does not identify a responsible entity to guide training implementation nor monitor compliance.

Notably, little evidence exists that IBT can change providers' clinical practice or patient outcomes.^29,37–41^ Some antibias interventions have been shown to reduce individuals' awareness of, level of, and/or motivations regarding their biases.^42–44^ However, antibias intervention effects are typically short-term, modest, have little effect on behavior, and do not occur consistently across settings.^45,46^ These patterns appear to be similar within and outside of health care contexts,^31,38,40^ reflecting an “irony that implicit bias training as currently envisaged might not be an effective or realistic approach to rectifying the negative effects of implicit bias.”^39(p.1458)^

In light of growing state and health care system IBT requirements,^34,47^ it is crucial to identify features of antibias interventions that may enhance their ability to improve care and patient outcomes.^29,47,48^ Engaging the lived experiences and insights of key stakeholders—in California, the Black women and birthing people whom the Act was designed to benefit, and the perinatal clinicians who will take IBT—is a critical way to access these insights.^49,50^ We describe these stakeholders' perspectives regarding the challenges and recommendations for impactful clinician IBT.

## Methods

With community collaborators and interdisciplinary researchers, we conducted a descriptive^51^ multimethods community-based participatory research (CBPR) study. Community collaborators (L.J., J.H-T., H.F.Y., B.P.), themselves IBT patient and advocate stakeholders, codesigned and provided sustained guidance on recruitment materials and strategy, data collection instruments, analysis, and presentation of results.

### Focus groups

We recruited individuals who received prenatal care, delivered a baby, and/or received postpartum care in a hospital in the San Francisco Bay area in 2019, 2020, or 2021. We advertised primarily via California birth equity-focused social media and fliers at large community clinics. We held five 90-min Zoom-platform focus groups (FGs) between August and November 2021 (3–7 participants per group). Participants received $75 for participation. A community collaborator with substantial CBPR expertise (L.J.) facilitated the FGs while a sociologist with substantial qualitative research expertise (S.B.G.) assisted and took analytic notes. The pair debriefed after each event to discuss content and data quality and finalize notes.

### Interviews

We recruited perinatal clinicians from two San Francisco Bay Area hospitals (community, safety-net), purposively sampling for variation in unit role and self-identified demographics. S.B.G. conducted semistructured in-depth interviews with each participant via phone (44–80 minutes; mean=56).

In both FG and interview engagements, we provided a written and verbal overview of the Act to ensure that participants had the same baseline knowledge of the legislation, its intent, and requirements (Supplementary SA). In addition, both formats of data collection focused on what factors would hinder or support impactful IBT, which we defined as IBT “that could improve care and clinical outcomes for Black women and birthing people.” We used a variety of prompts to thoroughly investigate these topics (Supplementary SB, and SC). S.B.G. took analytic notes in each engagement, specifically documenting what respondents said about factors that did or could hinder or support IBT's ability to advance birth equity. She verbally presented notes back to participants at the conclusion of each engagement to facilitate the correction, clarification, or addition of data.

With respondent permission, we digitally recorded FGs and interviews and had them professionally transcribed. (See Supplementary SD for additional methods information.) The UC San Francisco Institutional Review Board approved study activities; all participants provided verbal consent.

### Analysis

Following best practices, iterative combined inductive/deductive thematic analysis began and continued throughout data collection.^52,53^ The lead researcher added contextual and analytic notes to the notes participant-checked during interviews/FGs. Community collaborators (L.J., J.H., B.P., H.F.Y.) and S.B.G. discussed notes and transcript excerpts across numerous meetings to evaluate whether the study was capturing adequate data, to refine the data collection instruments, and to develop early familiarity with the data.

Upon conclusion of data collection, S.B.G., E.C., and S.Z. reviewed the full corpus of data for familiarity. In ATLAS.ti, we used a first round of codes to identify excerpts where participants described challenges, opportunities, or recommendations for implementing or achieving impactful IBT. In multiple meetings, SBG discussed segments of these data with community collaborators to check interpretation and to collectively identify and refine themes. S.B.G. developed a thematic coding scheme to characterize challenges and recommendations for implementing and achieving impactful IBT. S.B.G., E.C., and S.Z., and community collaborators reviewed the schema to evaluate its comprehensiveness and accuracy; collectively and iteratively refined its categories; and resolved disagreements via verbal and written discussion. S.Z. and E.C. applied these codes to the data in ATLAS.ti, which we used to extract relevant data and illustrative quotes. We present themes organized into five domains.

## Results

### Sample description

All FG participants (“patients”; *n*=20) identified as Black women, with 1 reporting additional racial identities (Table 1). Most patients were non-Hispanic/Latinx; had Medicaid insurance coverage; found it “somewhat” or “very” hard to pay for basic needs; and had a hospital-based delivery in 2020 to 2021. Interviewees (“clinicians”; *n*=20) were nurse midwives (*n*=6), physicians (*n*=6), registered nurses (*n*=5), or other staff (*n*=3). They self-identified as Black (*n*=4), multiracial (*n*=4), or white (*n*=12) women; two identified as Latinx or Hispanic women.

**Table 1.** Study Participant Characteristics

Patient characteristics	***n***	%
Race^a^		
Black or African American	19	95
Black or African American; Asian; Native Hawaiian or Pacific Islander	1	5
Latinx or Hispanic		
Yes	1	5
No	18	90
Prefer not to say	1	5
Gender identity		
Woman	20	100
Age in years		
20–29	3	15
30–39	16	80
40–50	1	5
Insurance type		
Medicaid (Medi-Cal)	12	60
Medicare	1	5
Private	6	30
Prefer not to say	1	5

**Provider characteristics**	***n***	**%**
Role		
CNM	6	30
Physician	6	30
RN	5	25
Lactation consultant	1	5
Social worker	2	10
Race^a^		
Asian, Native Hawaiian or Pacific Islander, other	1	5
Asian, white	1	5
Black or African American	4	20
White	12	60
White, other	2	10
Latinx or Hispanic		
Yes	2	10
No	17	85
Prefer not to say	1	5
Gender identity		
Woman	20	100

^a^ Participants were given the option to select “Other” as a racial designation. They were invited but not required to provide further information.

CNM, certified nurse-midwife; RN, registered nurse.

### Challenges to and recommendations for impactful IBT

Patients and clinicians raised many of the same factors that they believed threatened (challenges) or should be addressed to improve (recommendations) IBT effectiveness (Table 2). We highlight substantive differences when relevant. Identifying labels are provided to indicate the patient focus group (FG) or clinician interview (CA or CB), from which quotations are drawn (Table 3).

**Table 2.** Topics Represented in Respondents' Discussions of Challenges to or Recommendations for Effective Implicit Bias Training, by Respondent Subsample

	Patients	Clinicians
State law and policy		
Scope of trainees required to take IBT	x	x
Scope/intensity of IBT requirements	x	x
Accountability/enforcement of IBT	x	x
Funding	x	
IBT content, format and other qualities		
Content—Richness/nuance	x	x
Content—Connection to site	x	x
Content—Real patient stories	x	x
Content—Connection, relatability and credibility for providers	x	x
Format—Online self-administered	x	x
Format—Interactivity		x
Other—Application to practice and skills-building	x	x
Other—Frequency/regularity/continuity	x	x
Other—Limited impact on provider bias/behaviors	x	x
Health care facility IBT implementation		
Selection of trainers		x
Managing logistics		x
“Safety” of training environment	x	x
Use of data to inform and guide training approach	x	x
Health care facility environment of IBT		
Leadership decisions, commitment and communications re: IBT	x	x
Clinic culture and interpersonal dynamics		x
Accountability practices re: IBT and reductions in biased care	x	x
Opportunities for ongoing complementary antibias learning	x	x
Provider trainee commitment and behaviors		
Motivation/commitment to training	x	x
Recognition of own biases and need for change	x	x
Unintended effects	x	x

“x” denotes that one or more participants in the column subsample reported a challenge or recommendation related to the row topic.

IBT, implicit bias training.

**Table 3.** Recommendations for Impactful Clinician Implicit Bias Training (IBT): Illustrative Quotations from Patient and Clinician Stakeholders

State law and policy	
Scope of trainees required to take IBT	“They should make everyone train for that as far as nurses, clinicians, pretty much anyone in the medical field.” (FG04 #1) “Begin this training when they are getting their education… So it goes to everybody who's involved in this entire process from the lab technicians, the end people, the phlebotomists who are taking your blood, the nurse who's checking you in for your appointment… your OB. So everybody is getting the same training and it's across the entire span of your care and not just at this one stage of your care.” (FG05 #01)
Scope/intensity of IBT requirements	“Implement a time limit or a time frame on how much training should be done. You know, if you're doing a certain amount of hours of, you know, in order for them to really, really get it or really understand it. Like, not just a simple computer training for an hour and then that's it, you don't have to do it for another two years.” (FG03 #1)
Accountability/enforcement of IBT	“I think lawmakers could tie it to funding, right. So it's like if there's a lack of compliance, if hospitals aren't, like, you know, getting better results, that could have financial implications. I find that that's a great motivator.” (FG05#2) “Unless it's tied to something like performance standards, I think it's unlikely that any kind of a training really will drive the kind of change we need, right… I think people will go back to, like, business as usual unless it's, like, you know, “Oh, three strikes, I hit my three strikes and that means I'm on probation.” (FG05 #3) “If there was a law set or they pushed the law, everyone's going to listen to law… So I feel like if they implemented it and were strict on it, you would see less and less cases [of biased care]” (FG04 #1).
Funding	“Implement more funding so that aside from trainings, [providers] can be a little bit more educated on just the different mistreatments of African-American people or people of color in the hospitals.” (FG03 #1)

**IBT content, format, and other qualities**	
Content—Richness/nuance	“I would definitely put in, like, the history of Black people and medicine in this country so they can see it's not just something that might or might not be happening some places. I want—I would want them to understand that it's something that's systemic and it's, like, ingrained … and also it's probably in them and they don't realize it..” (FG02 #2) “Teaching people about the mistrust I think will be huge… Teaching the history of the mistrust of the Black community, and… the communities of color in general.” (FG02 #1)
Content—Connection to site	“Look at cases of people we took care of rather than have it be hypothetical… I think it would create—would definitely provide a mirror where we can really see how we in real-time are potentially causing harm. I think that it's just much more personal if it's someone that you took care of and you were, you or somebody you know, were involved in that person's care.” (CA07) “I think that would be really interesting if, like, specific to our hospital giving, like, specifics, so that it was real, that those [inequities] are happening.” (CA04)
Content—Real patient stories	“I really feel like hearing the narrative or the person that is impacted by implicit bias, bias and racism, I think those stories are sometimes more impactful and can create change than any, you know, one hour little training that the people are going to put together. Because sometimes you can—what's the word I'm looking for? Like, not associate that you're causing harm to real people… But say for example, like … five people who had been affected by implicit bias told their stories, but they didn't have to call the doctor out. But they told their story, [the doctor] would know, ‘Oh, that was my client.’ You know what I mean? ‘That was my patient.’ So I think it'd be more impactful that way, to know like, ‘Yes, doctor, you are causing harm to people, even though you might not have thought that you were doing so, but you've impacted someone in a negative way.” (CA01) “Tie it to patient examples or real-life experiences so that it would help put this theoretical thing in context. The example I gave with triage, I don't think anyone that would be involved with that would say, “I actively made this decision or said this thing about a patient because of their race,” but if confronted with that reality or shown that scenario and said, “How do you think that race would play into this?” I think might be a little bit more realistic… Sometimes more everyday, commonplace examples can be helpful.” (CB01)
Content—Connection, relatability and credibility for providers	“I would ask people a lot of times when they have felt like someone wasn't listening to them or you know, to start from a personal experience to personalize this.” (FG04 #3) “Training must get people to understand that they need a training.” (CA05) “There should be some type of way that providers can do some self-reflection and see like, what it is that they're doing in their own practice that could be—that implicit bias can be coming out that could be causing harm in that way.” (CA01) “You know, you can turn on a training and walk away and come back, you know. But if it grabs you, you're going to want to participate.” (CB03)
Format—Not using online self-administered	“if you had, like, during a skills day… where you could really really feel and sit down and actually take time and work within it. I think that is a better way than [online trainings]. We, honestly, we don't really—We're just trying to get through them as fast as we can because we're doing 10,000 other things at the same time.” (CB08) “It's such a terrible platform for learning, actually learning. People don't usually change just because they've interacted with a bullshit workplace education platform on the clock” (CB09)
Format—Interactivity	“I think that [impactful training] would allow for feedback, knowledge exchange, having other clinicians, having clinicians of color share what they've dealt with. I think just the sharing aspect of it because the computer, that's just kind of one-sided, right? …I think it would improve camaraderie, you have the clinic staff, everybody's there talking and I could say what I'd experienced, somebody else can say what they experienced.” (CA02) “You know, I will just say that any training that I've gone through that has really stuck with me is, like, role playing… If someone's put in that kind of situation where they're treated poorly because of what they look like, they're going to remember that.” (FG05 #1) “Having to, like, digest the course material and discuss it with other people can be challenging. But that's where a lot of times growth happens. And so having a module… where you're just, you know, you're reading it and then you're taking a test afterwards, isn't necessarily as helpful as, like, that processing with other people.” (CA04)
Other—Application to practice and skills-building	“Ideally, a training like that with such a sensitive subject would be like in person with protected time, where you get to like act out different scenarios. Like, [midwifery group] came and we did like an antiracist training… where we acted out scenarios, where someone says something and then you have to like think on your feet and adjust it and I find that much more impactful.” (CA09) “We did this one like OB emergency drill… and people moved through the room and so you weren't just, like, sitting… And there were those different speakers at each station doing a different skill thing. And so I think that, like, breaking it down like that could be really good…breaking it down and, like I said, applying it to patient care.” (CB06)
Other—Frequency/regularity/continuity	“At the rate that we're going, it should be like every 6 months, 6 to 12 months, like, because, like, 2 years is like you're going to take it one year and forget it 3 weeks later and just go back to, like, what you were doing previously.” (FG02 #1) “Did you say that this is something that has to be done every two years? (FG04 #3) Facilitator: Correct. “Come on, now. Every two years? This is—Implicit bias happens every day. This is a weekly training, not an every two year training.” (FG04 #3) “Exactly.” (FG04 #1) “Implicit bias training, I think, also needs to just be something that is just ongoing forever. For a long—that it's something that is required and ongoing, and right now.” (CA07)

**Health care facility IBT implementation**	
Selection of trainers	“Have somebody that's not from the hospital do – be the leader of the roundtable, and then they'll have times to really focus and get all their things dialed and be able to facilitate a conversation that is safe and that is – can get back on track… All the pitfalls of small-group leadership plus very confrontive topic, somebody that really has their kind of spiel and how they're going to do this and is comfortable with it, that could be I think a big boon.” (CB09) “Before the training gets rolled out there really needs to be—you really need to consult the people who are the most affected by it.” Interviewer: And when you're describing that are you picturing like people who would present the material or people who would develop the material? “Both really. If you're really going to be serious about it, it helps to come from the horse's mouth. It's just like a yoga teacher teaching somebody how to box. I mean like it needs to—there needs to be some credibility or some believability or some take it seriousness, something like that.” (CB05)
Managing logistics	Clinicians “have to definitely have a set-aside time where they are able to only do the training. And that would fail if they don't have that.” (CA04) “We are so overworked right now that, like, it's hard to find any extra time for anything. So [leadership] would have to allow us the time or, like, carve a certain amount of time or, like, have somebody cover us…” (CB08) “It's hard to leave the floor to do [trainings], but then people don't want to do it outside of work time, right. And so having an incentive. I mean, I don't want to say, I mean, yeah, in a way, that people are getting compensated for the training. I mean, everybody's time is valuable, you know.” (CB06)
“Safety” of training environment	“Emphasizing a non-judgment environment [would help]. I think that in medicine we've done a good job of shifting, reviewing mistakes for bad outcomes. It used to be that you're going to get – you're going to go down for this one… and we really shifted with a peer review sort of thing. Let's look at this bad outcome, and there's no blame. Let's just learn from it and understand how we can do better next time… I think developing some kind of a way [in IBT] of non-judgmentally fleshing out what people feel, what people – really looking at where what they feel comes from and non-judgmentally helping them with it.” (CB10) “I know there's some hard truths to be told in things like this but it has to be tactful enough to not make someone defensive… People shut down because they feel like something's directed towards them. So there needs to be some expert, some therapist or behavioral specialists or something like that will be able to just say maybe we could say this a different way just because it was more important just to get the point across. To not put people in defense.” (CB05)
Use of data to inform and guide training approach	“I think they should work on gathering data… Basically did this training change anything? … You know, reviews of, like, nurses' recommendations, doctors' recommendations, feedback that they get from their patients… Just, yeah, I worry if they're not gathering that information we'll never really know if it's working and then won't have any information to, like, make improvements if it is or if there's tweaks that need to be made.” (FG05 #6) “I think that they need to watch, maybe. Like, I want to say kind of like babysit the people that it didn't really reach. < laughs> And possibly put them through more training if they need to go through more training. But I think that the leaders need to be one, I think the leaders or whoever you choose in the hospital to be a leader, I think that they need to be a diverse group of people. And if they notice something off or if they notice somebody not really taking to this training, kind of help them, help them along with it. Like, give them scenarios, like real life scenarios.” (FG02 #1)

**Health care facility environment of IBT**	
Leadership decisions, commitment and communications re: IBT	“[Leaders should] keep talking about it. Help educate their other staff. Just make it an ongoing conversation. I mean, people might be like, “Okay, oh, my God, here she goes talking about it again.” But at least it's, like, I mean, I'm talking about it because it's important. I'm talking about it because I'm passionate about it and, you know, this is reality. Reality is is we, Black women go in, give birth and they're not coming out with their babies or vice versa. … So I would just say keep talking about it.” FG02#3 What leadership can do regarding providers who are not taking antibias work seriously: “Somebody can look at a screen, they can take the training and be like, “Blah, blah, I'm just going through a training.” But if you pull them to the side and say, “Hey, this is going on. That's going on.” A lot of times you just have to confront people head on.” (CA02)
Clinic culture and interpersonal dynamics	“Create a safe space for providers to talk, ask, interact… Maybe providing a safe space to have real conversations and to air real concerns. Yeah. I would like to ask questions and not be perceived as ignorant or racist for asking them, but we're all a little bit concerned.” (CB10) “I do think that being in person and being able to kind of form nonwork-related, like, have nonwork-related interactions and friendship could be really beneficial in fostering kind of like honest and vulnerable conversations.” (CA07)
Accountability practices re: IBT and reductions in biased care	“As long as there's like some kind of level after the training to hold people accountable [that] would be really important as well. Like, as far as there's clear steps as well on how to check in and make sure people are following protocol, also have clear steps for, like, people to be able to file a complaint and actually have them actioned… Also transparency as well… You guys could do the training but we also need to know that as patients that the training has been done and it's being implemented.” (FG01 #1) “What could possibly help is a penalty. Like, you know, how are you going to if you're caught doing the wrong thing to this patient, like, what are—What's the consequences going to be? … Penalty kind of makes people, like, “If we don't do this, we're going to get in trouble.” You know? “I might lose my job over mistreating a person of color.” And so therefore, all right, “Training says do this, then all right, I'm going to do this.” (FG03 #1) “For those who are less on the journey with [committed antibias work], we have to hold people accountable and that is hard and scary work, you know, where hierarchy is involved… Holding everyone accountable in a different kind of way than an eLearning module does.” (CA10) “How do you assess what the person got from the training? How likely are they to implement any of the training modules into their current practice and then according to the patients, like what does the before and after look like when a staff gets trained in implicit bias?” (CA08)
Opportunities for ongoing complementary antibias learning	“I just, I would like to see more, and more types of, you know, education—you know, maybe in person, maybe one on one… whatever it would take just to saturate a person's brain with these truths … I just think there just needs to be like more to counteract all of what's already in your head.” (CA06) “I'm not sure that a training just once a year will have a significant impact. I think what would have more of an impact is integrating training throughout the year and in various clinical settings and measuring that, not just a training once a year… Discussion of what it looks like to have dignity in pregnancy in childbirth—and that be a year-long discussion in various meetings or trainings outside of just the modules you do online … Internal trainings and discussions with leaders in the DEI space about how to address, diversity, equity and inclusion issues within the institution throughout the year… just having that set as an expectation. Then, you know, these sessions will happen throughout the year within various departments and not just once a year through a training because it's required by law. So better integrate it into the institution.” (CA08)

**Provider trainee commitment and behaviors**	
Motivation/commitment to training	“Just make sure the staff actually takes it seriously and not just like taking it just to say that they did it. Just taking it seriously and having an open mind taking the training.” (FG03 #3) “Getting people to understand how relevant this is and that it's a responsibility as a provider to have this training. Like, it's really as important as knowing how to whatever, you know, manage a hemorrhage… It really is.” (CB06)
Recognition of own biases and need for change	“I think to make change I think it usually works a little better if you're uncomfortable at the least, if that makes sense. Because if it's really well done, I hope that it would cause people to be a little introspective and pause for a moment and be like, “Hey, you know.” You see the title [of the training] and you're like, “Oh, I don't do that. I'm fine.” But hopefully if it's well done, it would cause people to think a little more… But not in a shameful way, because that doesn't work either. But in a thoughtful way.” (CB03) “People got to be aware of how they are affecting—how they are biased towards others. Because we can do all this training all we want and people …. probably won't take it serious because they think it's not, because they think they're not the problem… They have to be aware of their own actions and their own thinking, their own bias.” (FG01 #4)

“FG” denotes focus group participants: Black women who had been a patient in maternity care services. “CA” and “CB” denote interview participants from two facilities: multidisciplinary hospital-based perinatal clinicians.

### Domain 1: State law and policy

Both patient and clinician stakeholders referenced aspects of state law and policy in their discussions. Clinicians expressed concerns that the scope of providers required to complete IBT is insufficient, citing the breadth of nonperinatal providers who interact with pregnant individuals. Requiring only perinatal providers is “changing one last step” in the pregnancy journey (CB01). Both clinician and patient respondents recommended expanding requirements to include a broader range of health care providers and staff, including outpatient settings.

Both groups were concerned about the Act not requiring enough detail, specificity, or intensity of training—gaps that many participants addressed in recommendations for IBT design and implementation, below. Patients were particularly interested in making IBT more frequent and specifying the minimum number of hours of training to ensure a level of intensity and comprehensiveness.

Some stakeholders, particularly patients, identified the lack of enforcement or accountability mechanisms in the Act as a challenge. Patient participants recommended that IBT and antibias performance be linked to standards, penalties, or hospital funding, without which, IBT “won't change a thing” (FG05). Patients also recommended that funding be built into future policy to support more comprehensive antibias education.

### Domain 2: IBT content, format, and other qualities

Much of participants' discussion centered on challenges and recommendations for antibias training itself. Many participants expressed doubts that IBT could change clinician practice or deeply held biases (“You are who you are”; FG04), describing the influence of online IBT “like a drop in the bucket” (CA06). The majority of respondents also raised concerns that IBT was unlikely, on its own, to improve clinical outcomes for Black women and birthing people. Concerns focused on the training addressing a fraction of the problems that produce health inequities; that systemic factors, of which clinician bias is a part, will take generations to change; and that without systemic change, the training will have little power.

#### Content

Patients, and to a greater extent clinicians, shared concerns about IBT being superficial, repetitive, or overly simple: “It's different from fire safety. It's more nuanced” (CA09). Others felt trainings that were “generic” (CB10) and not specific enough to providers' patient population limited their impact. Patients and providers recommended that training provide a holistic treatment of racism, biases, mistrust, and inequities in U.S. medicine, although some felt that a better understanding of “racism as a whole” would have to precede this (FG02). Others suggested IBT include “powerful statistics” (CB03) that could demonstrate that racism, not race, underlies inequities. Many respondents recommended that IBT incorporate tailored information to connect it to the facility and community served (e.g., regarding patient experience, case studies, staff knowledge of racial disparities).

The majority of clinicians as well as some patients supported the use of real patient stories or narratives to make IBT impactful. Recommendations included narratives of what biased care feels like, of providers causing harm, and stories that ensured the experiences of Black women and birthing people, in particular, are “memorable” (CB08) and “real” (CA06) to IBT trainees.

Many clinicians felt IBT would be ineffective if their peers did not find it credible or relatable; if providers could not connect to the effort, they would not engage with it. Some had seen colleagues dismiss or become closed off to IBT. Clinician-respondents in particular recommended that training meet these challenges by fostering clinician reflection and introspection to understand bias in themselves (“make it personal,” CB06) and highlighting the ways reducing bias could improve clinical care.

#### Format

Participants from both groups, but particularly clinicians, highlighted the limitations of online self-administered training for changing biases and behavior. Multiple participants described such training as just a “box to check off” (e.g., FG04, CA05); one where distracted individuals could “click until it's over” (CA06) and not learn. “It's such a terrible platform for learning” (CB09). In response to this, many clinician-respondents recommended more interactive formats, featuring opportunities for colleagues to give and receive feedback, learn from each other, and be vulnerable and honest. Participants varied regarding what such sessions should look like (e.g., small group in-person versus online interactive sessions; interprofessional groups versus single-group trainings). A few clinicians also noted, however, that noninteractive trainings might better suit individuals for whom the stress or self-consciousness of interactive sessions would distract from their learning.

#### Other

Clinicians additionally discussed the limitations of IBT that lacked application to providers' day-to-day practice. Many expressed frustration that they were not learning strategies to use in clinic or hospital-based care. “Okay. I'm aware. So, what do I do from that?” (CA03). Both clinicians and patient respondents recommended that IBT integrate practical skills-building to make it “relevant, not theoretical” (CA10). Concrete examples included how to handle drug use conversations, patient disagreement on recommended treatment options, and other challenging clinic scenarios via strategies such as role playing, immersive/experiential teaching, and nonviolent communication. Regarding training frequency, multiple patient respondents felt that every 2 years would not be sufficient for changing behavior. Participants from both groups recommended training be implemented more frequently than dictated by law.

### Domain 3: Health care facility IBT implementation

Participants, particularly clinicians, raised a number of challenges and recommendations related to the implementation of IBT in hospitals. Clinician-respondents were concerned that facilities could, for example, adopt an ineffective curriculum or engage trainers who were insufficiently committed or credible. Clinician-respondents recommended that facilities engage trainers whom providers respect and/or perceive as appropriate for the role, including individuals from affected communities; others recommended professional external trainers. Patients, but particularly clinicians, recommended that facilities work to create a supportive environment for IBT. They called for “safe” (CB06), “nonjudgmental” (CB03) training spaces that allowed for participant privacy and were free of shaming and defensiveness.

Some clinicians felt that logistics, time pressures, and competing responsibilities—including numerous other trainings—would impede IBT or make it more burdensome. “We are so overworked right now… It's hard to find any extra time for anything” (CB08). Others reported that insufficient funding for antibias work exacerbated these problems and limited facilities' access to more impactful training options. Clinicians recommended facilities provide protected and paid time/coverage for IBT and ensure participant receipt of continuing education credits for it.

Some clinician-respondents expected that the lack of evaluation of IBT effects would limit its impact. Both patients and providers recommended that facilities use data (e.g., pre-/post-assessments; patient experience reporting) to evaluate if IBT is improving care and outcomes for affected communities; and/or to guide and refine future training.

### Domain 4: Health care facility environment of IBT

Patient and clinician participants also discussed challenges and recommendations related to the broader facility context in which training would take place. Some were concerned about leadership decisions, commitment, and communications about IBT. Some patients, for example, were concerned that hospital leaders could have the same biases IBT seeks to reduce, limiting leaders' support of training. Clinicians expressed concerns about rushed timelines and leaders who do not communicate the importance of IBT to staff. They recommended that leadership work to foster legitimacy and demonstrate commitment by allowing IBT appropriate time, fostering staff buy-in, and emphasizing its importance. Many participants recommended that facility leadership implement the policy- and training-focused recommendations described above (Domains 1–3).

Clinician respondents were particularly concerned about aspects of clinic culture and interpersonal dynamics that could limit the power or application of IBT. For example, several described that staff may not feel safe enough to let themselves be vulnerable in conversations about bias; or that hierarchies in the workplace inhibit discussion and critical feedback. Clinicians recommended the creation of spaces for antibias discussion and growth outside of training; fostering trusting relationships among staff; and developing a no-tolerance approach to bias and racism.

Primarily patients raised issues of accountability within the facility. They cited concerns that participation and change are unlikely without enforcement and that health care workers not required to take IBT may not participate. Patients in particular called for facilities to develop systems to be accountable for implementing IBT, and to hold themselves and providers accountable for providing unbiased care (e.g., vis-à-vis patient experience data, complaints). Other recommendations included patients being informed about providers' antibias training status and staff receiving supplemental training and, ultimately, penalties, if they continue to provide biased care.

Both groups shared concerns that antibias efforts could end up on the “back burner” at facilities (CB06). Recommendations included facilities providing digital and physical antibias reminders and creating regular unit-wide opportunities for complementary learning (e.g., discussions about bias and dignity in care; case reviews).

### Domain 5: Provider commitment and behaviors

Finally, participants expressed that IBT effectiveness would be limited for providers who are insufficiently motivated or committed to IBT, or who do not participate in a “wholehearted” way (CB07). Some felt that this was particularly likely for online-only IBT. Others were more concerned about colleagues’, teams’, or leaders' lack of commitment to antibias/antiracism change more broadly, which could dampen the impact of IBT in the unit. Patient and clinicians recommended providers work to take the training seriously and “have an open mind” (FG03). Participants described providers not recognizing their own biases or their need to change as barriers to effective IBT. “I think it's super easy to be dismissive of the training … “Yeah, yeah, the racist people of the world need this but, like, I don't” (CA05).

In addition to impeding training effectiveness, such denial can produce defensiveness among clinicians, which multiple respondents felt could cause them to “shut down” or react negatively to the training. To counter these challenges, many participants recommended that clinicians work to develop self-knowledge of their biases, for example, via reflection and discussion, described above.

Finally, a small number of patients and clinician stakeholders referenced potential unintended effects of IBT as a challenge. One FG participant, for example, described that providers “overeducated” in IBT could think that they know what patients need before patients tell them or could feel more entitled to “police” Black women (FG01). A clinician feared that IBT could contribute to burnout if it does not clearly serve the core mission of providing health care.

### Stakeholder expectations

**FIG. 1.**
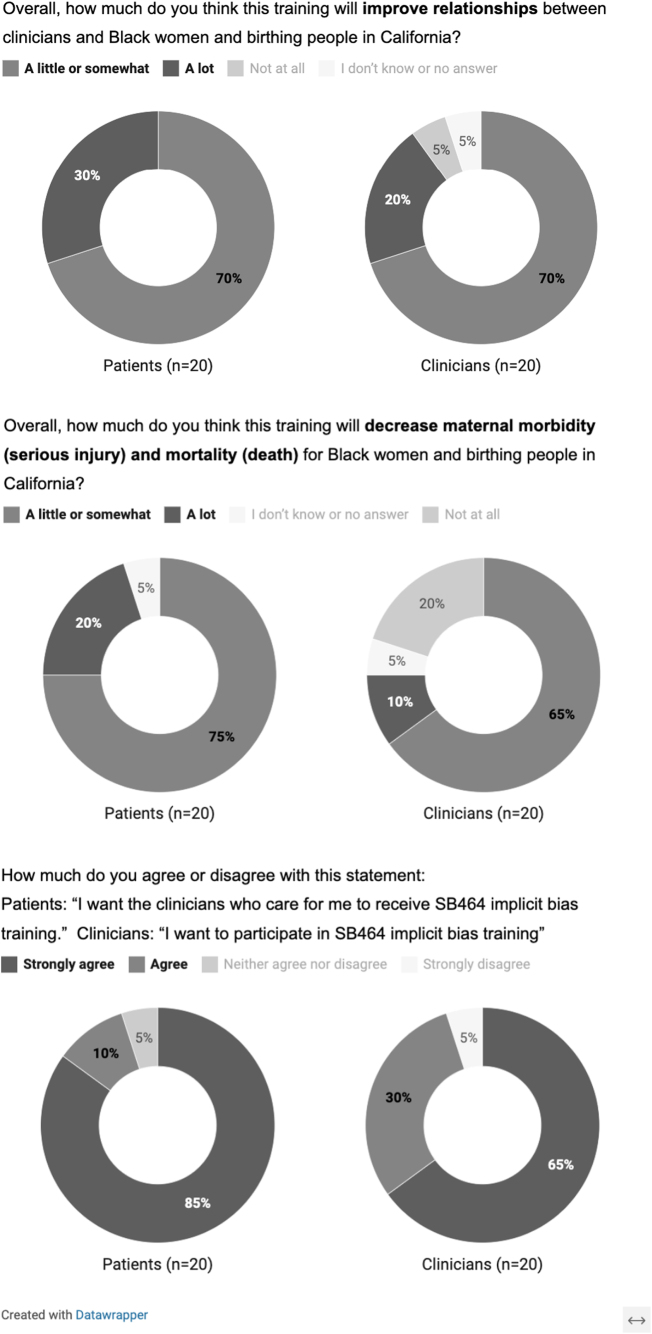
Patient and clinician opinions regarding the impact and desirability of implicit bias training. These graphics depict the proportion of respondents in each group that endorsed the presented survey responses to three opinion questions. Surveys were conducted at the conclusion of each data collection encounter.

Many participants expressed skepticism about IBT's effectiveness and felt that significantly reducing inequities in maternal care outcomes would only be achieved with multilevel interventions. Closed-ended questions asked at the end of these discussions reflected modest expectations. Most respondents expected that the training would decrease maternal morbidity and mortality, or improve relationships between clinicians and Black women and birthing people, “a little or somewhat” (Fig. 1). However, nearly all participants indicated that they wanted their provider to take IBT (patients) or that they themselves wanted to take it (clinicians). In the words of one participant, “I think the only outcomes [of IBT] would be either positive or nothing changes. And if it changed one Black woman's life, then it's all worth it” (FG02).

## Discussion

Patient and clinician stakeholders in the San Francisco Bay Area identified numerous challenges and actionable recommendations for IBT regarding aspects of (1) state law and policy; (2) IBT content and format; (3) health care facility IBT implementation; (4) health care facility environment; and (5) provider commitment and behaviors. We note that some factors presented as a challenge in one domain may be addressed by a recommendation in another. Patient and clinician insights overlapped substantially. Patients, however, focused more on aspects of state policy change than clinicians (e.g., IBT funding, intensity of training) and on the importance of accountability/enforcement in state policy and health systems.

Clinicians focused more on facility-based considerations (e.g., training logistics, clinic culture) and enhanced IBT features such as greater interactivity and use of real patient stories. Importantly, participants from both groups reported that IBT would improve the care and outcomes of Black women and birthing people only in concert with other system-focused equity interventions (e.g., workforce diversification; more accessible high-quality prenatal care).

Findings from this study align with some research and expert opinion on best practices for IBT. For example, participants underscored the importance of IBT that encourages critical reflection, builds skills, and avoids shaming.^30,47,54,55^ They also expressed the need for iterative, ongoing training to reduce implicit biases.^30,37,47^ Extending beyond typical IBT, they recommended evaluating the training's effect on patient experiences and outcomes^29,30,36,40,47^; improving the unit's interactional environment^30,46,56^; and complementary antiracism changes to the health care system.^29,33,36,40,57,58^

Our study yielded novel findings as well. Participants, particularly clinicians, stressed going beyond generic case studies to include real data from their patients, facilities, and colleagues. This may be a compelling way to foster perspective-taking – a component found to reduce bias in some populations.^32,38,54^ Patient stakeholders in particular recommended that IBT policy include robust accountability systems, involving people with lived experience of biased care, to strengthen the development, implementation, enforcement, and evaluation of trainings. These echo recommendations made by California birth equity advocates as well.^36^ The differences between our findings and typical IBT approaches likely reflect the unique wisdom of stakeholders and their focus on maternal health outcomes rather than on shorter-term attitudinal outcomes prevalent in IBT scholarship.

The findings have important and transferable^59^ implications for clinician IBT. First, state lawmakers have the opportunity to address many of the challenges stakeholders identified by integrating clear governance, monitoring, evaluation, and appropriate resources into IBT policy; and specifying more IBT features that stakeholders recommend (e.g., skills-building; site-specific content; live interaction). Specific action steps for policy-makers, codeveloped with multilevel IBT stakeholders and policy experts, are available elsewhere.^60^ Second, many California hospitals have utilized online, self-administered IBT curricula. Although thoughtfully designed,^61^ their format, intensity, and/or content do not currently match many stakeholder recommendations presented here. Even without a legal mandate, public and private funders, IBT curricula developers, and health care leaders should work collectively to integrate more stakeholder-endorsed components into future IBT offerings, then rigorously evaluate and refine these approaches.^41^ Mitigating qualities of health care settings known to exacerbate the effects of clinician bias (e.g., understaffing, time pressure) could additionally further antibias reduction.^32,54,62^

### Health equity implications

Evidence-based IBT is one of many interventions needed in our national effort to reduce maternal health inequities.^63–65^ As with all health equity interventions, it will be most successful if designed with the robust and ongoing input of the individuals closest to the problem.^49,50,66,67^ For clinician IBT, this means engaging the Black women and birthing people from communities disproportionately burdened by maternal health inequities—to assess whether the intervention can meaningfully improve their care experiences and outcomes—and the clinicians, to assess whether the intervention will be feasible, sustainable, and impactful in their workplaces. With the growth of clinician IBT requirements, it will be crucial for stakeholder wisdom to guide future efforts.

### Strengths and limitations

This study centered stakeholders in both its research and operations. Study leadership and collaborators included community stakeholders, and the study sample represents diverse members of key IBT stakeholder groups, thereby capturing an important range of views and experiences. However, study respondents were based in a single region—California's Bay Area—and did not include ABC clinicians. Preliminary survey research has indicated that current and former health care workers outside of our study region largely support recommendations reported here.^68^ However, future studies should thoroughly explore stakeholder priorities and realities in other settings.

## Conclusion

Patient and clinician stakeholders identified numerous challenges to IBT improving care and clinical outcomes for Black women and birthing people. Recommendations spanned several domains, reflecting the multilevel work that will be required to advance maternal health equity. Stakeholder-identified challenges and recommendations represent crucial insights for the development and implementation of health equity interventions. Lawmakers and health system leaders should leverage these and other stakeholder insights in IBT decision-making.

## Supplementary Material

Supplemental data

## Data Availability

The data underlying this study cannot be shared publicly due to ethical concerns. Interested parties may contact the study PI, Sarah Garrett (sarah.garrett@ucsf.edu) to inquire about selective access.

## Supplementary Material

Supplementary SA

Supplementary SB

Supplementary SC

Supplementary SD

## Abbreviations Used

ABC: alternative birth centers
CNM: certified nurse-midwife
FG: focus group
IBT: implicit bias training
RN: registered nurse
