# Supplementary material for: Challenges and Opportunities for Clinician Implicit Bias Training: Insights from Perinatal Care Stakeholders
Source: Health Equity. 2023 Sep 13;7(1):506–19. doi: 10.1089/heq.2023.0126 (PMC10507933; doi:10.1089/heq.2023.0126)
Supplement: Supplemental data [file Suppl_Data.pdf]

SUPPLEMENTARY MATERIAL for “Challenges & opportunities for clinician implicit bias training: Insights from perinatal care stakeholders”

Supplement A. Overview of Senate Bill 464 – MEND handout

Supplement B. Focus Group Guide

Supplement C. Interview Guide

Supplement D. COREQ Summary of Study Methods

Supplement A. Overview of Senate Bill 464's/"The California Dignity in Pregnancy and Childbirth Act.

This handout was provided to all clinician participants prior to, and overviewed during, the research interview. The similar patient-facing version, provided to participants prior to and overviewed during the focus groups, is available here.

WHAT IS SB 464 AND WHAT IS IT SUPPOSED TO DO?

Black women are 3 to 4 times more likely to die from pregnancy-related causes than white women.[1] Racial bias is a factor that impacts the quality of care that Black women receive during and after pregnancy and childbirth. **SB464 is a new state law that aims to decrease racial disparities, decrease provider bias, and make more data available about maternal deaths in California.**

Effective as of January 1, 2020, the law requires:

1. Hospitals and alternative birth centers (ABCs) **must provide implicit bias training for perinatal providers** every 2 years.
2. Updates to the California Electronic Death Registration System to reflect the U.S. Standard Death Certificate and **more detailed information about pregnancy status in relation to time of death.**
3. The California Department of Public Health (CDPH) **must track and publish maternal mortality data by race, ethnicity, and region.**
4. **Hospitals must provide more information to every patient upon admission about their right to be free of discrimination and where they can report discrimination in their health care.**

HOW DOES IT WORK?

1. **Implicit Bias Training**
   - ⇒ Hospitals and alternative birth centers must provide implicit bias training for providers involved in the perinatal care of patients.
   - ⇒ Perinatal providers at these facilities must complete an initial training, then a refresher course every 2 years. Hospitals will issue certificates of completion.
   - ⇒ Training must be evidence-based and address:
     - Provider implicit bias, its effects, and steps to decrease it at individual and institutional levels.
     - Topics such as: health inequities, barriers to care and inclusion, cultural identity, provider-community relationships, communication, and reproductive justice (see page 2).
   - ⇒ **The law does not say how long the training must be, how it should be taught, or how the law will be enforced.**
   - ⇒ **The law does not necessarily apply to workers who provide prenatal or postpartum care only outside of hospitals or ABCs.**

2. **Death Certificate Reporting**

[1] Petersen EE, Davis NL, Goodman D, et al. Racial/ethnic disparities in pregnancy-related deaths — United States, 2007–2016. *MMWR Morbidity & Mortality Weekly Report*. 2019;68:762–765.

⇒ The California Electronic Death Registration System will **more precisely report** when a patient was pregnant in relation to time of death.

3. **CDPH Publication of Data**
   ⇒ CDPH will **track and publish information on pregnancy-related deaths**, and present data by region and race/ethnicity.
4. **Patient Education**
   ⇒ Hospitals are required to provide patients with the following written information on admission:
   - **That patients have a right to be free of discrimination**.
   - **How to file a complaint** with CDPH, CA Department of Fair Employment and Housing, or the Medical Board of California **if they have experienced discrimination**.

THE 10 REQUIRED TOPICS FOR SB464 IMPLICIT BIAS TRAINING

1. Identification of previous or current **unconscious biases and misinformation**.

2. Identification of personal, interpersonal, institutional, structural, and cultural **barriers to inclusion**.

3. Corrective **measures to decrease implicit bias** at the interpersonal and institutional levels, including ongoing policies and practices for that purpose.

4. **Information on the effects**, including, but not limited to, ongoing personal effects, **of historical and contemporary exclusion and oppression of minority communities**.

5. Information about **cultural identity** across racial or ethnic groups.

6. Information about **communicating more effectively** across identities, including racial, ethnic, religious, and gender identities.

7. Discussion on **power dynamics** and **organizational decision-making**.

8. Discussion on **health inequities within the perinatal care field**, including information on **how implicit bias impacts maternal and infant health** outcomes.

9. Perspectives of diverse, local constituency groups and experts on particular **racial, identity, cultural, and provider-community relations issues in the community**.

10. Information on **reproductive justice**.

Created by the MEND Study, UC San Francisco (2021)

Supplement B. Focus Group Guide, with associated probes, for Patient Stakeholder Participants in the MEND study

1. **Facilitator introductions & informed consent**

2. **Participant experiences & context**

    - *Please introduce yourself and describe your experience when you were last at a hospital for something related to pregnancy or birth.*

    Thank you. For the rest of the discussion, we're going to be asking you questions to learn what is and isn't working for Black women and birthing people at hospitals, how to make it better, and how a recent law may or may not help. As we talk through all of this, please keep in mind your experiences, what you want, and what you think needs to happen to improve maternity care for Black women and birthing people.

3. **Implicit bias in healthcare / Overview SB464 & requirements**

    California's new law, Senate Bill 464 / The California Dignity in Pregnancy and Childbirth Act, requires clinicians who care for pregnant and birthing people to take implicit bias training. The goal of these new laws is to improve health outcomes for Black women and birthing people.
    For our conversation, we will define implicit bias as unconscious prejudices, attitudes, and stereotypes that clinicians may have about certain people or groups. Implicit bias training is a training designed to reduce or weaken these biases.

    [Overview SB464 handout]

- *Does anyone have questions they want to discuss about SB464? Or first impressions to share before we move on to some specific questions?*

4. **Participant experiences with bias or racism in healthcare**

- *Has provider bias or racism affected the care you have received? How?*
- *Has bias or racism in the health system affected the care you have received?*
- *How?*

5. **Patient perspectives on how/why IBT would or would not change their care, outcomes; and how it aligns with what they value**

Will implicit bias training…
- *Change relationships between clinicians & Black women and birthing people at your hospital? Why or why not?*

- *Change health outcomes for Black women and birthing people at your hospital? Why or why not?*
- Probes:
    - *How could it fail?*
    - *How could it work (or "succeed") to improve care and care outcomes?*
    - *Are there positive effects it might have? What are they?*
    - *Are there negative effects it might have? What are they?*
    -
- *How do you want your doctor to treat you?*

[Administration of Qualtrics-based closed-ended questions: Participant level of support for the 10 topics in SB464's implicit bias training requirements]

- *Are there are any other topics that you'd want to add to the list of implicit bias training topics? Topics that would support the goal of improving care and outcomes for Black women and birthing people?*

6. Improving care and outcomes for Black women and birthing people

- *What do you recommend for how to improve care & outcomes for Black women and birthing people?*
- *What advice to you have for <u>hospital leaders</u>…*
    - *… about implicit bias training?*
    - *… about improving outcomes for Black women and birthing people?*
- *What advice to you have for <u>state lawmakers</u> about improving outcomes for Black women and birthing people?*

7. Reflections & checking

- *Have we missed anything in today's discussion?*

[Administration of Qualtrics-based closed-ended questions regarding expectations & support for SB464 training.]

[Facilitators present analytic notes and interpretation back to participants.]

- *Does anyone want to add information that we missed? Or correct something? We want to make sure we truly understand your ideas and experiences.*

Thank you so much for sharing your stories and wisdom with us.

Supplement C. Interview Guide, with associated probes, for Perinatal Clinician Stakeholder Participants in the MEND study

1. **Interviewee context**
    - *In a couple sentences please tell me about a typical day for you in the hospital. What you do and who do you see?*
    - *About how long have you been at this hospital?*
    - *How long have you been a clinician?*

2. **Implicit Bias Training**
    - *Today we're going to be talking primarily about implicit bias training (IBT) for perinatal care providers.*
    - *Are you familiar with this term? What do you know about IBT?*
    - *For the purposes of this conversation, we will consider implicit bias to be unconscious prejudices, attitudes, and stereotypes that individuals may have about certain people or groups. IBT tries to reduce these biases.*
    - *What are a few words or feelings that come to mind when you think about IBT?*
    - *Have you heard about the California bill SB464? What do you know about it?*
        - *In advance of our meeting I sent an overview of the new state policy, SB464. I'll take a moment to review key aspects of it with you…* [Review handout]

3. **Feelings about the new legislation**
    - *How do you feel about SB464's IBT requirement?*
        - *In your experience [or when you imagine yourself doing it] what do you think has been [will be] a difficult or uncomfortable part of this training?*
    - *Has your hospital told you how you and your colleagues should fulfill the IBT that SB464 requires?*
    - *Will you [continue to] participate in these trainings? Why or why not? What will your involvement look like?*

4. **How responsive clinicians think SB464 is to the needs of Black women and birthing people and the factors that could improve their clinical outcomes**
    - *When you think about this hospital, do you feel clinician bias affects clinical outcomes for Black women and birthing people? How?*
    - *Do you think racism—either individual or structural—affects care outcomes for Black women and birthing patients? How?*
    - *I'm going to ask you two survey-type questions and then ask you to talk through your answers:*
        - *How much do you think that the SB464-mandated IBT will improve clinicians' relationships with Black women and birthing people at your hospital?*
        - *How much do you think that the SB464-mandated IBT will improve the clinical outcomes of Black women and birthing people at your hospital*

- *Have you heard about a time when a Black women or birthing patient had a negative experience or clinical outcome at your hospital? What do you think caused it?*

5. **Feasibility and effectiveness of SB464**
    - *Reflecting on your past experiences with IBT or similar training, what are some ways you feel it did not "work"? How or why did it not improve patient care or outcomes?*
    - *When you think about IBT for your hospital, what could go wrong? What are the challenges? In what ways might it fail to improve the care or outcomes of Black women and birthing people?*
    - *Reflecting on your past experiences with IBT, what are some ways you feel it "worked"? How did it improve patient care or outcomes?*
    - *When you think about IBT at your hospital, what might help it to improve care for Black women and birthing people? What would this look like?*
        - *What could your hospital or colleagues do to help it succeed?*
        - *Is there anything else you would you recommend?*
    - *How, if at all, do you feel that these trainings have affected you or your practice? How do you feel it has affected your colleagues? The labor & delivery floor overall?*
    - *If you were going to measure (or evaluate) the effects of IBT, what would be important to measure?*
    - *I'd like to check the notes I took about what we're considering the challenges, opportunities, and recommendations of IBT with you…* [Check interpretation]

6. **Recommendations to improve care outcomes for Black women and birthing people**
    - *Thinking more broadly here, when you think about labor & delivery leadership, hospital leadership, or state lawmakers, what recommendations do you have for improving clinical outcomes for Black women and birthing people?*
    - *Do you have any other advice for hospital or labor & delivery leadership as they begin to implement these IBT trainings?*
    - *What advice do you have for hospitals or labor & delivery leadership who want to improve clinical outcomes for Black women and birthing people?*
    - *What advice do you have for state lawmakers who want to improve clinical outcomes for Black women and birthing people?*

7. **Closed-ended questions**
    - *Please think about the IBT that Senate Bill 464 (SB464) requires perinatal providers to do.*
    - *Overall, how much do you think this training will improve relationships between clinicians and Black women and birthing people in California?*
      □ *A lot* □ *Somewhat* □ *A little* □ *Not at all*

- *Overall, how much do you think this training will decrease maternal morbidity (serious injury) and mortality (death) for Black women and birthing people in California?* □ *A lot* □ *Somewhat* □ *A little* □ *Not at all*

- *How much do you agree or disagree with the following? I want to participate in SB464 IBT* □ *Strongly disagree* □ *Disagree* □ *Neither agree or disagree* □ *Agree* □ *Strongly agree.* □ *I'm not sure*

8. Additional relevant information or recommendations

- *What else should I have asked you about this topic?*
- *Is there anything we haven't addressed that you think is important to discuss?*

Supplement D. Summary of Study Methods per Consolidated Criteria For Reporting Qualitative Studies (COREQ).[1]

The methods description immediately below refers to data collection for both the interview and focus group branches of the MEND study (Multi-Stakeholder Engagement with State Policies to Advance Antiracism in Maternal Health). When information varies by format, it is presented separately within the category.

No	Item	Guide questions/description
**Domain 1: Research team and reflexivity**		
Personal characteristics		
1.	Data collector	Focus group facilitator: Doula and birth justice worker with expertise in group facilitation<br>Interviewer: PhD sociologist with expertise in qualitative methods
2.	Credentials	See above.
3.	Occupation	Focus group facilitator: Doula, founder of Black Women Birthing Justice, community research collaborator<br>Interviewer: Assistant professor, researcher
4.	Gender	Women
5.	Experience and training	Focus group facilitator: Extensive experience respectfully eliciting birth stories and experiences from individuals from historically minoritized communities, particularly Black women.<br>Interviewer: Doctoral-level training in sociology with intensive expertise with collecting and analyzing qualitative data.
Relationship with participants		
6.	Relationship established	The relationship commenced with study recruitment and lasted for the duration of the focus group or interview.<br>We later sent information about study findings, including an online town hall, to former participants.

No	Item	Guide questions/description
7.	Participant knowledge of the data collector	Participants understood the study team's goals, which were the goals of the study: To learn about challenges to and recommendations for clinician implicit bias training as required by CA Senate Bill 464 (SB464), with the goal of improving care and clinical outcomes for Black women and birthing people.
8.	Data collector characteristics	Focus groups: The facilitator (LJ) identified as a Black woman and doula and was visible on screen. The PI and note-taker (SBG) identified as a white woman and was visible on screen. We expected that the presence of a white woman in the focus group might constrain discussion. The facilitator addressed this proactively and encouraged the participants to be as honest and critical as they would without a white person on the call.<br><br>Interviewer: Beyond her interest to contribute to scientific knowledge, the interviewer identified and reported no personal characteristics that we believe would contribute to bias in the interview encounter. The interviewer clarified that she had no formal relationship to the institution where clinician respondents worked. The interviewer has a first name common among white American women of her age group. When the topic of racial identity arose in interviews, the interviewer (re)identified herself as white. No interviewees expressed surprise at this disclosure.
**Domain 2: study design**		
Theoretical framework		
9.	Methodological orientation and Theory	We ground this descriptive qualitative study[2] in subtle/critical realism, acknowledging (a) subjectivity in respondents' and analysts' understandings and descriptions of reality and the socially-constructed nature of reality; and (b) the existence of a reality that analysts can work toward understanding through thoughtful study design, data collection, and triangulation in analysis.<br><br>Additionally, the study draws on tools and concepts from implementation science to try to understand and characterize the adoption, adaptation, and potential impact of novel antibias and/or antiracism interventions.

No	Item	Guide questions/description
Participant selection		
10.	Sampling	Focus group participants:<br><br>We recruited individuals who were English-speaking, over age 18, identified as Black or African American, and lived and had received hospital-based perinatal care in the San Francisco Bay Area. As the number of candidates that enrolled approximately matched the number of participants we needed, we did not employ a purposive or quota-based sampling approach.<br><br>Interview participants:<br><br>Purposive sampling of clinician respondents who provide hospital-based perinatal care in Northern California, in order to interview individuals in different roles in perinatal care units.<br><br>Clinicians were eligible if they self-identified as providing perinatal care in a hospital; one or more of the facilities where they worked was one of our study sites; were over 18 years old; and identified they could participate in an English-language interview.<br><br>Interview participants: As more individuals expressed interest than we had time or funding to interview, we conducted purposive sampling by inviting individuals representing:<br>• Varied training backgrounds and clinical roles (physicians [MDs], registered nurses [RNs], certified nurse midwives [CNMs], social workers [SWs], International Board Certified Lactation Consultants [IBCLCs], and medical assistants [MAs]).<br>• Varied demographic characteristics in order to attempt to maximize diversity of race, gender and ethnicity in the sample. The candidate pool unfortunately had little variation in ethnicity or gender, which is reflected in our final sample.<br>• Varied dates of contact with our study: We expected that individuals most enthusiastic about and interested in antibias/antiracism interventions would be among those who responded most quickly to our study invitations. We therefore extended the recruitment period over months, and across multiple waves of study advertisement/recruitment, in order

No	Item	Guide questions/description
		to also try to recruit individuals who were likely less passionate or interested in these topics. We believe this gave us access to a broader range of perspectives and experiences than if we had recruited only the first/early phase of volunteers.
11.	Method of approach	How were focus group participants approached?<br><br>We distributed study advertisements and information via widely-read California-based birth equity social media, such as Voices4BirthJustice and the social media channels of UC San Francisco's Preterm Birth Initiative. Interested candidates were invited to contact the study directly via phone, text, or email; or to use a QR code link to access a short screening survey that provided additional information about the study and collected their contact information. Most candidates contacted us using the link/screening survey.<br><br>How were interview participants approached?<br><br>After receiving permission from departmental leaders at each facility, the principal investigator (SBG) sent an email with information about the study, eligibility criteria, and study activities, which departmental leadership forwarded to perinatal staff and clinician email lists. Interested candidates could email the PI directly or could follow a link to an online screener and contact form.<br><br>The PI contacted them directly to provide more information and invite them to participate. Candidates were given the option of scheduling directly with the PI or using a scheduling website (Calendy) to self-schedule. Nearly all used Calendly.
12.	Sample size	20 Black women who had a recent hospital birth<br><br>20 perinatal clinicians
13.	Non-participation	Focus group participants:<br><br>37 individuals expressed interest by contacting us, most via the online screening survey website.

No	Item	Guide questions/description
		6 of those individuals were not eligible for the study because:<br>• They were not based in the San Francisco Bay Area (4)<br>• They contacted us after our recruitment window closed (2).<br><br>For the 31 who were eligible, the study co-investigator/lead community collaborator (LJ) reached out to provide more information about the study and informed consent, and to confirm interest.<br><br>20 of 31 candidates ultimately participated in the focus groups, yielding a participation rate among candidates of 65%.<br><br>Proximate reasons for non-participation included the investigator's inability to make initial contact with the candidate (4), or to contact the candidate for subsequent focus group scheduling (7), potentially indicating candidates' lack of continued interest or lack of time.<br><br>Interview participants:<br><br>39 individuals expressed interest via email or online screener and provided their contact information to the PI.<br><br>6 of those 39 individuals were not eligible for the study because:<br>• they did not work in a hospital-based perinatal setting (4);<br>• they worked at a hospital where MEND had not initiated recruitment (1); or<br>• they contacted the study after recruitment had concluded (1).<br><br>Of the 33 who were eligible, the PI employed purposive sampling to invite 28 to participate in an interview.<br>20 of 28 invited individuals were ultimately interviewed, yielding a participation rate among invited candidates of 71%.<br><br>Proximate reasons for non-participation included PI's inability to make contact with the candidate for scheduling (7), potentially indicating candidates' lack of continued

No	Item	Guide questions/description
		interest or lack of time. 1 individual self-scheduled an interview but did not attend and could not be contacted to reschedule.
Setting		
14.	Setting of data collection	Focus groups:<br><br>Focus groups were conducted via Zoom, using personal computers, tablets, or mobile phones. Participants were asked to take the call in a private location. Participants were offered a digital device to use for the focus group if needed; none elected to use this option.<br><br>Interviews:<br><br>Semi-structured in-depth interviews were conducted via phone. Participants were asked to take the call in a private location.<br><br>Data collection at one site preceded the hospital's implementation, meaning that respondents discussed expected challenges and benefits based on their experience of their facility and other trainings. At the other site, data collection overlapped with the implementation of training, so respondents were able to represent both their expectations and reality of the trainings.
15.	Presence of non-participants	Focus groups:<br><br>Facilitators occasionally observed non-participants in participants' presence, typically related to childcare needs. These individuals did not participate in focus group discussions.<br><br>Interviews:<br><br>The interviewer observed no non-participants; interviewees reported none.
16.	Description of sample	Focus groups were conducted August 2021 to November 2021. Focus group participants (n = 20) identified as Black women, with 1 reporting additional racial identities.

No	Item	Guide questions/description
		Interviews were conducted August 2021 to March 2022. The interview sample was diverse in self-identified race and clinical role; all identified as women (n = 20).<br><br>See Table 1 for more information.
Data collection		
17.	Interview guide	The focus group facilitator used a semi-structured focus group guide that had been developed and pilot-tested prior to initiating focus groups.<br><br>The interviewer used a semi-structured interview guide that had been developed and pilot-tested prior to interviews.<br><br>For both approaches/samples: To enhance study rigor and the validity of the data, the interviewer/facilitator (SBG) took detailed notes on key topics during the interviews/focus groups and presented her understanding of these data back to the respondents at the end of the research encounter for the respondent to clarify, correct, and/or affirm. These key topics focused on specific challenges and recommendations for clinician implicit bias training.<br><br>See Supplement B for focus group guide; Supplement C for interview guide.
18.	Repeat data collection	There were no repeat focus groups or interviews.
19.	Audio/visual recording	With respondent permission we digitally recorded all focus groups/interviews and had them professionally transcribed. Identifying information was removed before analysis.
20.	Field notes	Field notes were taken during the focus groups and interviews and included among study data.<br><br>The interviewer/facilitator (SBG) created structured analytic case summaries for each research encounter that reflected the respondents’ perspectives and experiences on

No	Item	Guide questions/description
		challenges, opportunities, and recommendations for clinician IBT, among other topics.
21.	Duration	Focus groups lasted approximately 90 minutes.<br><br>Interviews ranged from 44 – 80 recorded minutes, with the majority between 50 and 60 minutes (mean = 56).
22.	Data saturation	The research team discussed data saturation, specifically whether we had collected enough data to answer our main questions, at multiple points throughout the project. The insights for which we sought to reach saturation concerned domains of challenges to and recommendations for clinician implicit bias training. We feel we have reached saturation on these points.
23.	Transcripts returned	We did not return transcripts to participants. However, as described above (#17), the PI/interviewer checked her understanding of key points with respondents.
**Domain 3: analysis and findings**		
Data analysis		
24.	Number of data coders	For the indexing of topics and the coding described in the attached paper, there were 3 coders: EC, SZ, SBG.
25.	Description of the coding tree	The major codes relevant to this paper were:<br>• Recommendations for improving IBT<br>• Challenges to IBT being impactful<br>• Codes describing the level or domain of the factor discussed (e.g., the learners; the training; state policy)<br><br>We used these codes to extract data for iterative inductive analysis described below.
26.	Derivation of themes	Phase 1. Starting during data collection, overlapping groupings of the research team reviewed transcripts, excerpts, and analytic notes to facilitate familiarity with the data. Focusing on challenges to and recommendations for

No	Item	Guide questions/description
		impactful clinician implicit bias training, team members iteratively identified, discussed, and documented the topics and early patterns they detected.<br><br>After the conclusion of data collection, EC, SZ, and SBG established high-level topic codes to use to label excerpts of the transcripts related to challenges and recommendations, and the domains in which they occurred (see #25). They used these to excerpt data for a subsequent phase of focused thematic data analysis to understand manifestations of specific types of challenges and recommendations within these domains. They reread excerpts numerous times, wrote analytic notes[9], and reviewed notes and output collectively.<br><br>Over the course of multiple discussions, SZ, EC, SBG inductively developed and refined their description and organization of themes regarding challenges and recommendations for effective IBT. We looked for nuance and disconfirming evidence to assess and hone our interpretation.[10,11] We resolved the small number of differences of interpretation via group discussion and refinement of concepts.<br><br>The coding team presented their analysis, the organization of domain-specific themes, and a wide range of data to community advisor collaborators (LJ, JH, BP) who agreed with and affirmed their analysis.<br><br>An audit trail was maintained throughout to capture analytic processes and decisions.
27.	Software	Atlas.ti and Excel
28.	Participant checking	Participant checking of key insights, including all factors participants raised as hindering or potentially supporting IBT effectiveness, occurred during the focus groups and interviews as described above (#17).
Reporting		
29.	Quotations presented	*Were participant quotations presented to illustrate the themes / findings? Was each quotation identified? e.g. participant number*

No	Item	Guide questions/description
		We include participant quotations to illustrate recommendation-focused themes and findings in Table 3. Quotes from participants are identified by a code denoting participant number (e.g. 01), source of data (patient focus groups [FG], clinician interviews [IV]), and clinician participant site (site A, site B)
30.	Data and findings consistent	*Was there consistency between the data presented and the findings?*<br><br>Yes
31.	Clarity of major themes	*Were major themes clearly presented in the findings?*<br><br>Yes
32.	Clarity of minor themes	*Is there a description of diverse cases or discussion of minor themes?*<br><br>Yes

REFERENCES

1. Tong A, Sainsbury P, Craig J. Consolidated criteria for reporting qualitative research (COREQ): a 32-item checklist for interviews and focus groups. *Int J Qual Health Care*. 2007;19(6):349-357. doi:10.1093/intqhc/mzm042

2. Rendle KA, Abramson CM, Garrett SB, Halley MC, Dohan D. Beyond exploratory: a tailored framework for designing and assessing qualitative health research. *BMJ Open*. 2019;9(8):e030123. doi:10.1136/bmjopen-2019-030123

3. National Coalition for Hospice and Palliative Care. Clinical Practice Guidelines for Quality Palliative Care, 4th edition. Published October 31, 2018. Accessed October 25, 2018. https://www.nationalcoalitionhpc.org/ncp-guidelines-2018/

4. “Mini-mental state”. A practical method for grading the cognitive state of patients for the clinician - PubMed. Accessed June 18, 2021. https://pubmed-ncbi-nlm-nih-gov.ucsf.idm.oclc.org/1202204/

5. Nasreddine ZS, Phillips NA, Bédirian V, et al. The Montreal Cognitive Assessment, MoCA: a brief screening tool for mild cognitive impairment. *J Am Geriatr Soc*. 2005;53(4):695-699. doi:10.1111/j.1532-5415.2005.53221.x

6. Creswell JW. *Qualitative Inquiry and Research Design: Choosing among Five Approaches*. 2nd ed. Sage Publications, Inc; 2006.

7. Abramson CM, Dohan D. BEYOND TEXT: USING ARRAYS TO REPRESENT AND ANALYZE ETHNOGRAPHIC DATA. *Sociol Methodol*. 2015;45(1):272-319. doi:10.1177/0081175015578740

8. Gale NK, Heath G, Cameron E, Rashid S, Redwood S. Using the framework method for the analysis of qualitative data in multi-disciplinary health research. *BMC Medical Research Methodology*. 2013;13(1):117. doi:10.1186/1471-2288-13-117

9. Miles MB, Huberman AM. *Qualitative Data Analysis: An Expanded Sourcebook*. 2nd ed. Sage Publications, Inc; 1994.

10. Braun V, Clarke V. Reflecting on reflexive thematic analysis. *Qualitative Research in Sport, Exercise and Health*. 2019;11(4):589-597. doi:10.1080/2159676X.2019.1628806

11. Braun V, Clarke V. What can “thematic analysis” offer health and wellbeing researchers? *International Journal of Qualitative Studies on Health and Well-Being*. 2014;9(0). doi:10.3402/qhw.v9.26152
